# The C-terminal domain of RD3 enables accumulation of retinal membrane guanylyl cyclase (RetGC) in photoreceptor outer segment

**DOI:** 10.1016/j.jbc.2026.113277

**Published:** 2026-06-23

**Authors:** Elena V. Olshevskaya, Igor V. Peshenko, Alexander M. Dizhoor

**Affiliations:** 1 Pennsylvania College of Optometry, Drexel University, Pennsylvania, USA; 2 Department of Neurobiology and Anatomy, College of Medicine, Drexel University, Pennsylvania, USA

**Keywords:** calcium binding proteins, cyclic GMP (cGMP), GCAP, GC-E, guanylate cyclase (guanylyl cyclase), photoreceptor, RD3, RetGC, retina, retinal degeneration, signal transduction, vision

## Abstract

Retinal degeneration-3 protein (RD3) plays a dual role in photoreceptors – prevents their degeneration by suppressing aberrant activity of retinal membrane guanylyl cyclase (RetGC) in the inner segment and enables photoreceptor function by facilitating delivery of RetGC to the outer segment. Parts of RD3 structure supporting its dual function were evaluated *in vivo* using deletion mutants of a human RD3 transgenically expressed under the control of rod opsin promoter in *Rd3*^*−/−*^ mouse rods lacking endogenous RD3. The human RD3 truncated after Gly^148^ or Arg^158^ not only inhibited RetGC activation by the guanylyl cyclase activating protein (GCAP) *in vitro* but also prevented rapid degeneration of the RD3-deficient rods in transgenic mice. However, these deletion mutants did not restore RetGC trafficking in *Rd3*^*−/−*^ rods to the outer segment or normal rod function. Extending RD3 polypeptide to Ser^170^ restored RetGC accumulation in the outer segment of rescued *Rd3*^*−/−*^ rods and enabled their photoresponse. These findings indicate that the RD3 serves as a ‘ski lift’ for RetGC produced in the inner segment, in which Arg^158^–Ser^170^ region of RD3 mediates coupling of the RetGC:RD3 complex to intracellular protein trafficking, while its α-helical core N-terminal to Gly^148^ binds RetGC and suppresses its aberrant activation by GCAP in the inner segment in order to prevent degeneration of photoreceptors.

Retinal guanylyl cyclase (RetGC) plays a critical role in rod and cone physiology by generating the phototransduction second messenger cGMP. The predominant isozyme of RetGC, RetGC1 (encoded by *GUCY2D* in humans and *Gucy2e* in rodents), produces most of the cGMP utilized in rod and cone phototransduction (1, 2, 3, 4, 5, 6). The activity of RetGC in photoreceptor outer segments maintains a fraction of cyclic nucleotide gated-channels (CNGCs) in the open state, thereby partially depolarizing the photoreceptor membrane in the dark. After illumination, phosphodiesterase 6 (PDE6) hydrolyzes cGMP, resulting in closure of CNGCs and hyperpolarization of photoreceptors, which initiates the process of visual perception (reviewed in (1, 2, 7, 8, 9)).

Two types of regulatory proteins control RetGC activity – guanylyl cyclase activating proteins (GCAPs) and retinal degeneration-3 protein (RD3) (reviewed in (1, 2, 10, 11, 12)). GCAPs regulate RetGC in the outer segment by mediating negative Ca^2+^ feedback based on changes in free Ca^2+^ concentration between light and dark. Mg^2+^-liganded GCAPs accelerate RetGC activity after illumination, when the influx of Ca^2+^ through CNGC**s** stops, and shut-off RetGC in the dark when Ca^2+^ influx through CNGCs resumes, converting GCAPs to their Ca^2+^-liganded state. The GCAP-mediated regulation of RetGC by negative Ca^2+^-feedback contributes to shaping rod and cone photoresponses and their adaptation to background light (13, 14, 15, 16, 17).

In contrast, retinal degeneration-3 protein (RD3) regulates RetGC activity in the photoreceptor inner segment (18, 19), where it plays two major roles essential for photoreceptor physiology and survival. RD3 is required for the delivery of RetGC from the inner segment, where it is produced, to the outer segment (12, 20, 21), where it enables normal photoreceptor function. In addition, high-affinity binding of RD3 to RetGC prevents its aberrant activation by GCAPs (18, 19, 22), thereby playing a critical role in the survival of rods and cones. Aberrant activation of RetGC by GCAPs during transit from the inner segment results in a rapid, severe degeneration of RD3-deficient rods and cones in human patients with Leber’s congenital amaurosis type 12 (LCA12) and in the mouse *rd3* strain (23, 24); reviewed in (1, 12).

The RD3 structure includes a four-α-helical bundle, primarily formed by the N-terminal half of the molecule, and an extended flexible C-terminal portion (Fig. 1*A*) (25). Our previous studies (26, 27) identified two clusters of residues in the α-helical core of RD3 that are critical for its high-affinity binding to RetGC (Fig. 1*A*). In contrast, no residues essential for high-affinity binding were found in the unstructured C-terminal region of the molecule. We hypothesized that, instead of binding to RetGC, the C-terminal part of RD3 participates in trafficking of the RD3:RetGC complex, helping RetGC to reach the outer segment. In this study, we present *in vivo* evidence supporting this hypothesis and identify the ‘trafficking signal’ on RD3 required to direct RetGC to the outer segment.

**Figure 1:**
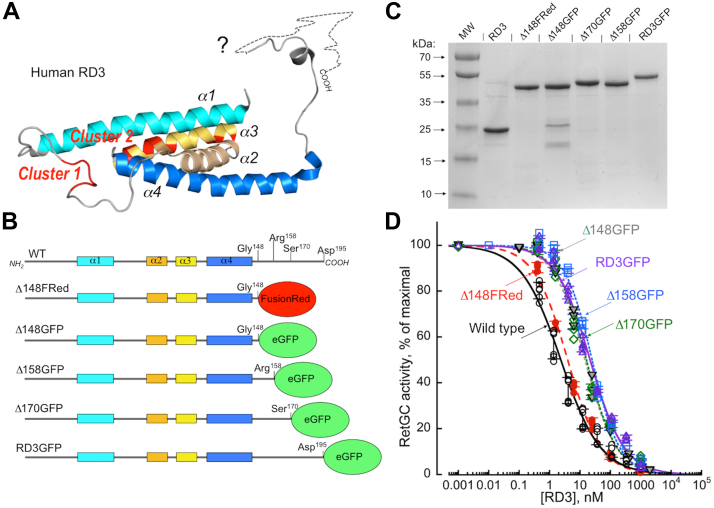
*A*, The human RD3 structure consists of a four alpha-helical bundle and an unstructured C-terminal domain (25). The alpha-helical core contains two clusters of residues (*red*), located in the loop between helices α1 and α2 (Cluster 1) and on helix α3 (Cluster 2), that are most critical for the ability of RD3 to bind and inhibit RetGC1 (27). The function of the unstructured C-terminal part of the molecule was undetermined. *B*, the RD3 constructs designed to evaluate the RD3 function *in vivo*. Deleted portions of human RD3 were substituted at the indicated positions by fluorescent proteins tags, FusionRed in Δ148FRed or eGFP in Δ148GFP, Δ158GFP RD3, and Δ170GFP. In RD3GFP, the tag was attached to a full-length RD3 (18). *C*, coomassie-stained 4-20% gradient SDS PAGE of non-tagged human RD3 and tagged RD3 constructs purified from *E*. *coli*. *D*, the RD3 variants suppress the activity of RetGC1:GCAP1 complex *in vitro*. Recombinant RetGC1 activity in HEK293 cell membranes was assayed in the presence of 1.5 μM Mg^2+^GCAP1 at the indicated concentrations of RD3 (**○**, *black circles*), Δ148FRed (●, *red filled circles*), Δ148GFP (**▽**, *gray triangles*), Δ158GFP (▪, *blue squares*), Δ170GFP (^◇^, *green diamonds*), and RD3GFP (^△^, *magenta triangles*); the symbols represent independent measurements using different preparations of HEK293 membranes containing RetGC1. Averaged data from independent trials, normalized as % of maximal activity in each case, were fitted assuming a sigmoidal function, *A = 100/(1+([RD3]/EC*_*50*_*)*^*h*^*)*, where *A* is the RetGC activity, *[RD3]* – concentration of RD3, *EC*_*50*_ – RD3 concentration causing twofold reduction of the activity, and *h* – Hill coefficient). The respective EC_50_ values were 2.3, 3.9, 14.6, 25.1, 16.3, and 21.5 nM; error bars – SD.

## Results

### Truncated RD3 tagged with fluorescent proteins binds to and inhibits RetGC1 *in vitro*

**Figure 2:**
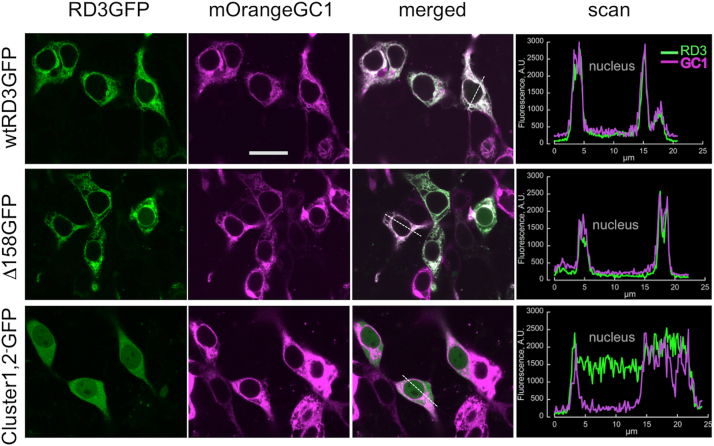
**Binding of RD3 to RetGC1 requires an intact RD3 helical bundle core but not the C-terminal part.** Cell-based assay of RD3GFP (*green*) co-localization with mOrange-tagged RetGC1 (*magenta*) co-expressed in HEK293 cells. Wild type RD3GFP (*top row*) and Δ158GFP RD3 (*middle row*) both effectively co-localize with RetGC1 in endoplasmic reticulum membranes and do not diffuse into the nucleus, whereas full-length RD3GFP with point mutations inactivating both Cluster 1 and Cluster 2, W62A/L63R/R101A/Q102L (27), remains diffusely distributed through the cytoplasm and the nucleus in the presence of RetGC1; the rightmost panels in each row show the representative distribution of RD3 and RetGC1 fluorescent tags across the cell; bar – 20 μm.

To study the role of RD3 *in vivo* in transgenic mice, we generated a series of RD3 deletion mutants in which different portions of their C-terminal region were substituted with GFP or monomeric FusionRed (FRed) (28) fluorescent protein tags (Fig. 1*B*). Fluorescent tagging of deletion mutants was required to more reliably verify their presence in transgenic mice *in vivo* because truncation of RD3 can drastically reduce the efficiency of its detection by anti-RD3 antibody (20). The tagged deletion mutants, Δ148GFP, Δ148FusionRed, Δ158GFP, and Δ170GFP, expressed in *E*. *coli* (Fig. 1*C*), retained the ability of RD3 to uncouple the GCAP1:RetGC1 complex *in vitro* at submicromolar concentrations (Fig. 1*D*), regardless of the length of the RD3 polypeptide in the tagged construct. The protein tag had some effect on binding affinity, as the apparent affinity in Δ148FRed was higher than in Δ148GFP (Fig. 1*D*) and similar to that of untagged RD3. However, the effective concentrations (EC_50_) of all tagged RD3 constructs that reduced twofold the activity of the GCAP1:RetGC1 complex formed in the presence of 1.5 μM Mg^2+^GCAP1 were in the nanomolar range similar to that of full-length RD3GFP (Fig. 1*D*). It is important to emphasize that, despite a moderate reduction in apparent affinity for RetGC1 in comparison with the untagged RD3 *in vitro*, RD3GFP fully substitutes endogenous RD3 *in vivo*, as previously demonstrated by its ability to completely rescue *Rd3*^*−/−*^ mouse rods from degeneration and restore their function (18, 29). Similar to the previously tested full-length RD3GFP and Δ148RD3GFP (26), Δ158RD3GFP also showed strong co-localization with RetGC1 in a cell-based assay (Fig. 2). In contrast, full-length RD3GFP harboring point mutations in two clusters that define the high-affinity RetGC-binding sites in the RD3 α-helical core (Cluster 1 and Cluster 2, Fig. 1) located upstream from the C-terminal truncated portion of RD3 showed weakened co-localization with RetGC1 (ref. 27 and Fig. 2). Because all deletion mutants were able to effectively bind to and inhibit RetGC1 *in vitro*, we subsequently used Δ148FRed, Δ158RD3GFP, and Δ170RD3GFP for expression in RD3-deficient transgenic mice.

### C-terminal RD3 deletion mutants rescue RD3-deficient rods from rapid degeneration

**Figure 3:**
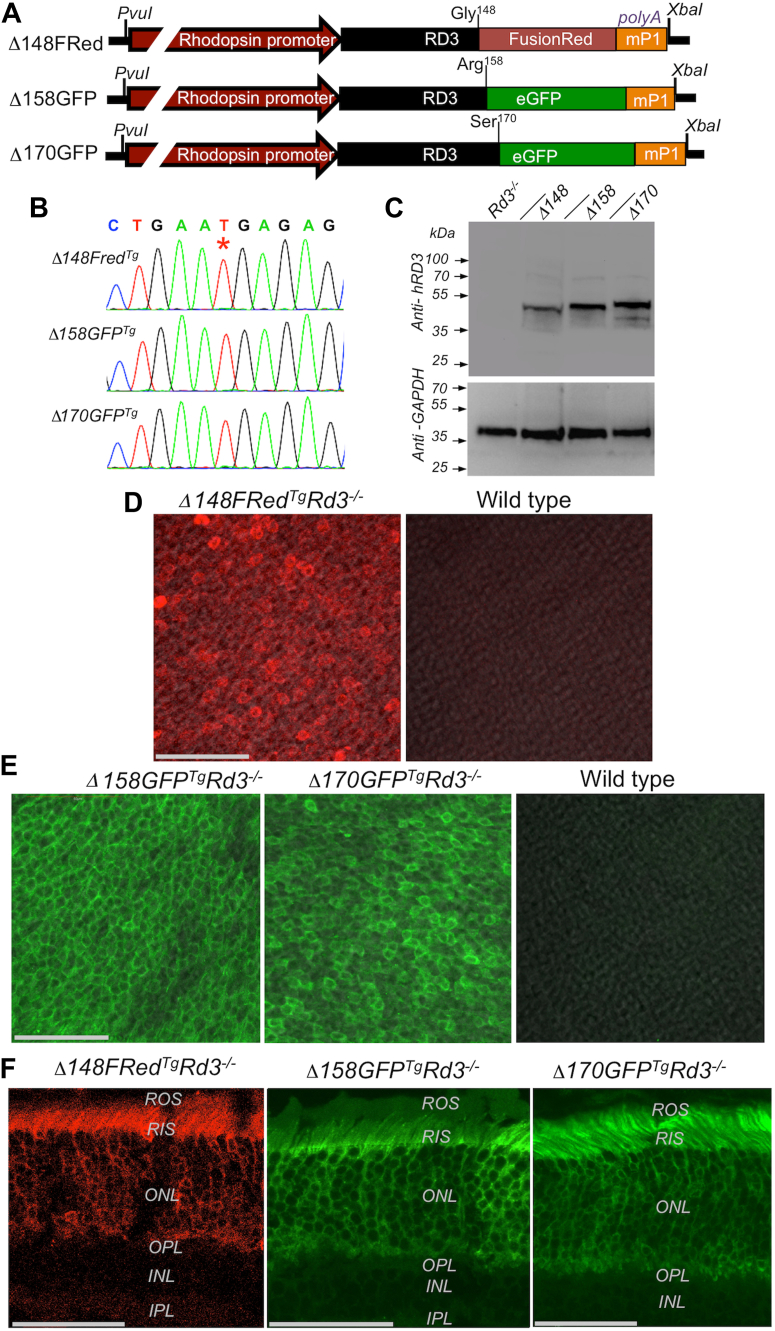
**Transgenic expression of C-terminally truncatedRD3mutants in mouse retinas.***A*, schematics of DNA constructs for transgenic expression of Δ148FRed RD3, Δ158GFP RD3, and Δ170GFP RD3 in mouse rods. All constructs contained a 4.2 kb rhodopsin promoter (49), the respective RD3 cDNA, and a mouse protamine gene exon containing polyadenylation signal. The constructs excised using PvuI/XbaI digestion were used for random insertion into the DNA of fertilized mouse eggs. *B*, representative PCR verification of the *Rd3*^*−/−*^ genotype in established *Δ148FRed*^*Tg*^*Rd3*^−/−^ (*top*), *Δ158GFP*^*Tg*^*Rd3*^−/−^(*middle*), and *Δ170GFP*^*Tg*^*Rd3*^−/−^ (*bottom*) lines using Sanger sequencing of mouse tail DNA: the thymidine (∗) replacing cytosine confirms homozygous c.319C→T transition in the third exon of mouse *Rd3* gene causing the *rd3* nonsense mutation R100ter (23) in all these lines. *C*, Western immunoblot of *Rd3*^−/−^, *Δ148FRed*^*Tg*^*Rd3*^−/−^, *Δ158GFP*^*Tg*^*Rd3*^−/−^, and *Δ170GFP*^*Tg*^*Rd3*^−/−^ retinas probed with anti-human RD3 (*top panel*) antibody. The same membrane was then stripped and re-probed as described in Experimental procedures for glyceraldehyde 3-phosphate dehydrogenase (GADPH, *bottom panel*). *D–F*, confocal images of the photoreceptor layer expressing truncated RD3 transgenes; bars – 50 μm. *D* and *E*, Flat-mount views of the retina superimposed on DIC images. *D*, FusionRed fluorescence in *Δ148GFP*^*Tg*^*Rd3*^−/−^ (*left*) in comparison with the wild type control (*right*). The red fluorescence in both cases was acquired using the same excitation and emission acquisition settings. *E*, Flat-mount view of eGFP fluorescence in *Δ158GFP*^*Tg*^*Rd3*^−/−^ (*left*) and *Δ170GFP*^*Tg*^*Rd3*^−/−^ (*middle*) *versus* the wild type control (*right*). The fluorescence in all cases was acquired using the same excitation and emission acquisition settings. *F*, cross-sections of *Δ148FRed*^*Tg*^*Rd3*^−/−^ (FusionRed fluorescence), *Δ158GFP*^*Tg*^*Rd3*^−/−^, and *Δ170GFP*^*Tg*^*Rd3*^−/−^ (eGFP fluorescence); *INL*, inner nuclear layer; *IPL*, inner plexiform layer; *ONL*, outer nuclear layer; *OPL*, outer plexifirm layer; *RIS*, rod inner segment; *ROS*–rod outer segment.

Δ148FRed, Δ158GFP, and Δ170GFP were expressed in mouse rods under the control of the rod opsin promoter using the same approach as we previously used for expressing full-length RD3-GFP (18) (Fig. 3*A*). The established transgenic lines were subsequently outbred to the *rd3/rd3* mouse strain (*Rd3*^*−/−*^) made congenic with C57B6J background (16, 18, 26) until all the lines were homozygous for the c.319C→T transition (*rd3* mutation, ref. (23)) detected by Sanger sequencing (Fig. 3*B*). Although immunoreactivity of RD3 is reduced by deletion of the C-terminus and cannot be used for direct comparison of the levels of expression, each RD3 deletion variant transgenically expressed in *Rd3*^*−/−*^ retinas was detectable by Western blot (Fig. 3*C*). Their fluorescent tags used to ensure the transgene expression were clearly visible in a large population of rods (Fig. 3, *D* and *E*) and mostly present in their inner segments and cell bodies, although some Δ170RD3GFP was also detectable in outer segments (Fig. 3*F*).

**Figure 4:**
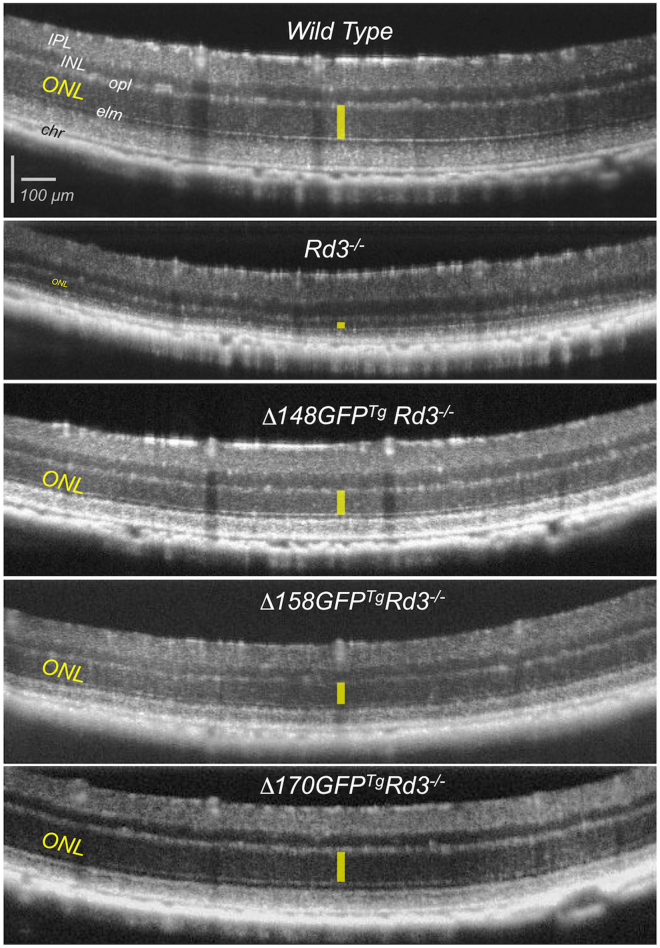
**Preservation of photoreceptor layer in mouse *Rd3*^*−/−*^ retinas expressing C-terminally truncated mutants of human RD3.** Representative *in vivo* retinal OCT images in (*top to bottom*) wild type, *Rd3*^*−/−*^, *Δ148FRed*^*Tg*^*Rd3*^−/−^, *Δ158GFP*^*Tg*^*Rd3*^−/−^, and *Δ170GFP*^*Tg*^*Rd3*^−/−^ mice aged 4 months. The thickness of the outer nuclear layer (ONL) is marked in *yellow*; *chr*, choroid; *elm*, external limiting membrane; *INL*, inner nuclear layer; *IPL*, inner plexiform layer; *opl*, outer plexiform layer. See Experimental procedures for detail.

**Figure 5:**
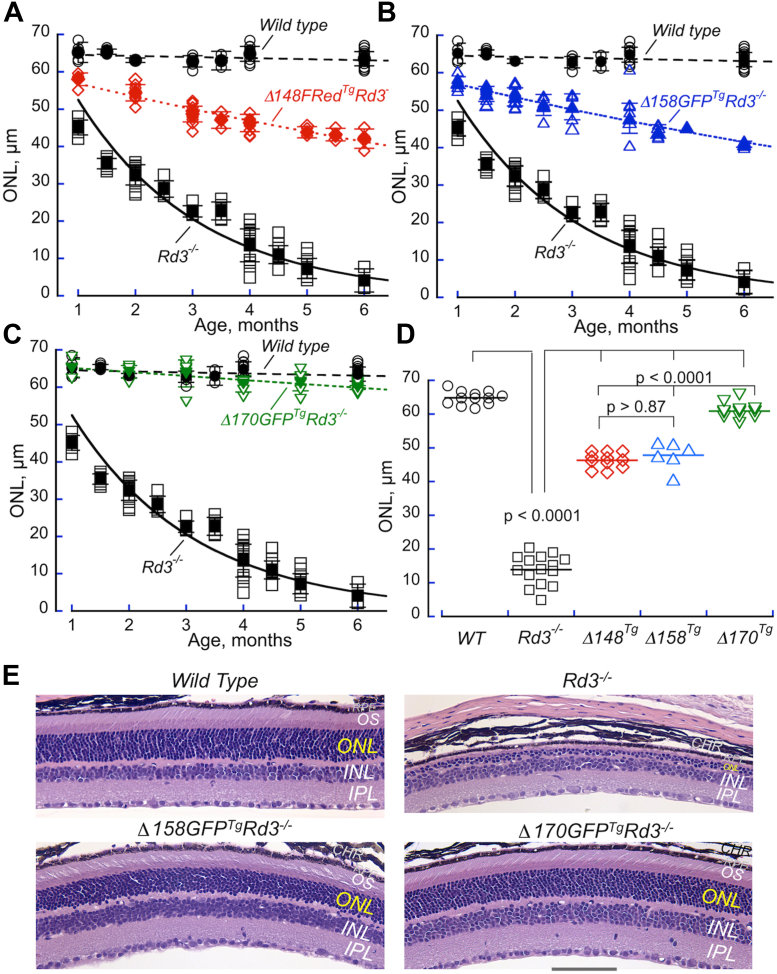
**Truncated RD3 delays progression of *Rd3*^*−/−*^ retinal degeneration.***A–C*, ONL thickness measured by OCT at different ages in wild type (○●) and Rd3^−/−^(□▪) in comparison with Δ148FRedTgRd3^−/−^ (◇◆, *red diamonds*) (*A*), Δ158GFPTgRd3^−/−^ (▵▲, *blue triangles*) (*B*), or Δ170GFPTgRd3^−/−^ (▿▼, *green triangles*) (*C*); open symbols – individual animals, filled symbols – mean average ± SD; the averaged data are fitted assuming exponential decay. *D*, ONL thickness measured by OCT in wild type (○), *Rd3*^*−/−*^(□), *Δ148FRed*^*Tg*^*Rd3*^−/−^ (^◇^, *red diamonds*), *Δ158GFP*^*Tg*^*Rd3*^−/−^ (^△^, *blue triangles*), and *Δ170GFP*^*Tg*^*Rd3*^−/−^ (^▽^, *green triangles*) mice aged 4 months; the *p* values are from an ANOVA/Tukey HSD *post hoc* test. *E*, representative cross-sections of the retina in wild type, *Rd3*^*−/−*^, *Δ158GFP*^*Tg*^*Rd3*^−/−^, and *Δ170GFP*^*Tg*^*Rd3*^−/−^ aged 5 months; RPE – retinal pigment epithelium, OS – outer segment layer; bar – 100 μm.

The thickness of the photoreceptor layer in the transgenic retinas of living mice at different ages was compared with that of *Rd3*^*−/−*^ and wild-type age-matched littermates using optical coherence tomography (OCT) (Fig. 4). The outer nuclear layer (ONL), comprised of photoreceptor nuclei (97% rod/3% cone (30)), was already 30% thinner than normal in *Rd3*^*−/−*^ mice at 1 month of age. It further degenerated rapidly by half within ∼2 months and lost more than 80% of photoreceptor nuclei by 6 months of age. All three truncated RD3 mutants effectively slowed the progression of degeneration (Figs. 4 and 5, *A–C*). A much larger fraction of photoreceptor nuclei remained after 4 months in both *Δ148FRed*^*Tg*^*Rd3*^−/−^ and *Δ158GFP*^*Tg*^*Rd3*^−/−^ (71% and 74%, respectively) and was ∼94% normal in *Δ170GFP*^*Tg*^*Rd3*^−/−^, in stark contrast to only 21% in *Rd3*^*−/−*^ at that age (ANOVA/Tukey HSD, *p* < 0.0001 in all three cases) (Fig. 5*D*). There was no significant difference between *Δ148FRed*^*Tg*^*Rd3*^−/−^ and *Δ158GFP*^*Tg*^*Rd3*^−/−^ (ANOVA/Tukey HSD, *p* > 0.87) (Fig. 5*D*). The histological features of the retina were also dramatically improved as compared to those in *Rd3*^*−/−*^, where the remnants of photoreceptors at that age did not form a discernible layer of outer segments (Fig. 5*E*).

### Rod function in Rd3^−/−^ mice harboring deletion mutants of RD3

**Figure 6:**
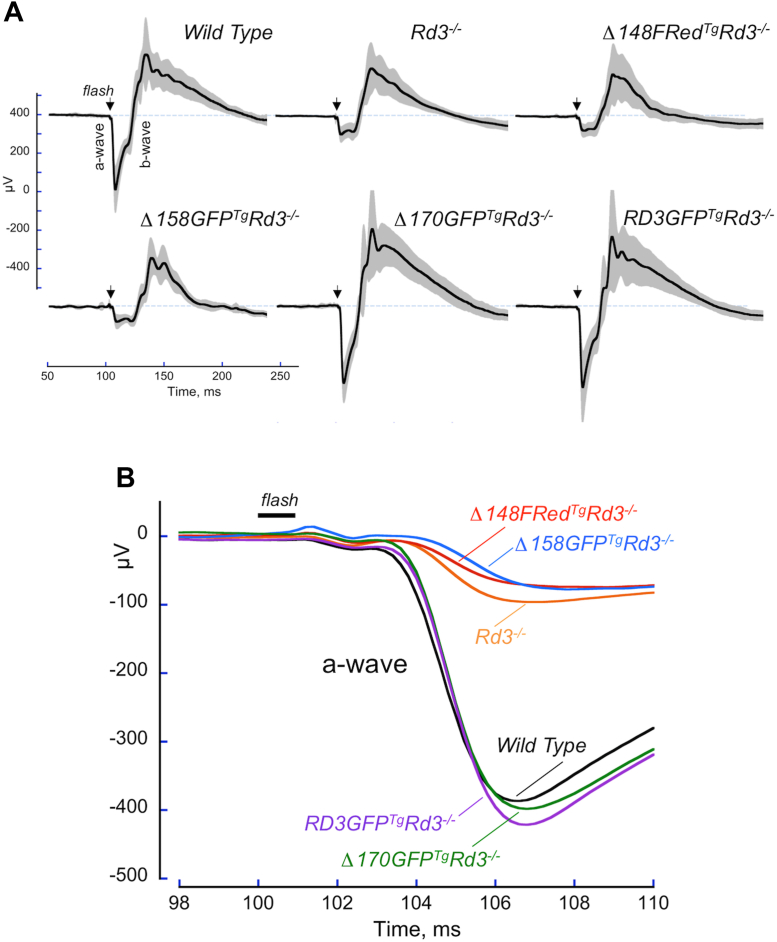
**Restoration of *Rd3*^*−/−*^ rod function by recombinant RD3 requires its C-terminal region, Arg^158^–Ser^170^.***A*, dark-adapted ERG responses to a bright (∼0.5 × 10^6^ photoisomerizations/rod) flash in wild type, *Rd3*^*−/−*^, *Δ148FRed*^*Tg*^*Rd3*^−/−^, *Δ158GFP*^*Tg*^*Rd3*^−/−^, *Δ170GFP*^*Tg*^*Rd3*^−/−^, and *RD3GFP*^*Tg*^*Rd3*^−/−^mice aged between 1 and 1.5 months; solid lines – mean, shaded areas – SD. *B*, averaged a-waves traces in wild type (*black*), *Rd3*^*−/−*^ (*orange*), *Δ148FRed*^*Tg*^*Rd3*^−/−^(*red*), *Δ158GFP*^*Tg*^*Rd3*^−/−^(*blue*), *Δ170GFP*^*Tg*^*Rd3*^−/−^ (*green*), and *RD3GFP*^*Tg*^*Rd3*^−/−^ (*purple*) mice; flash duration – 1 ms.

Rod function in transgenic mice aged 1 to 1.5 months was tested using dark-adapted full-field electroretinography (ERG) (Fig. 6*A*). The amplitude of the ERG a-wave produced primarily by hyperpolarizing mouse rods (31) in response to a bright (∼0.5 × 10^6^ R^∗^ rod^−^^1^) flash was reduced fourfold in RD3-deficient retinas, down to 94 ± 42 μV (mean ± SD) from 382 ± 117 μV in wild type (ANOVA/Tukey HSD, *p* < 0.0001) (Fig. 6*B*). Despite much better preservation of rods in *Δ148FRed*^*Tg*^*Rd3*^−/−^ and *Δ158GFP*^*Tg*^*Rd3*^−/−^ mice in comparison with *Rd3*^−/−^ (Fig. 5), their ERG a-wave amplitudes remained heavily suppressed (70 ± 35 μV and 74 ± 35 μV, respectively), when compared to wild-type mice (*p* < 0.0001) (Fig. 6*B*). The rescued rods of the two hybrid genotypes remained virtually as dysfunctional as *Rd3*^*−/−*^ (*p* = 0.973 and 0.994, respectively). In contrast, rod photoresponse was dramatically improved in comparison with *Rd3*^−/−^ in *Δ170GFP*^*Tg*^*Rd3*^−/−^ (397 ± 103 μV, *p* < 0.0001) (Fig. 6*B*). The ERG response in *Δ170GFP*^*Tg*^*Rd3*^−/−^ was restored as effectively as in rods rescued by full-length RD3GFP (18) (419 ± 184 μV, *p* = 0.988) and did not significantly differ from the wild-type control (*p* = 0.999).

### The length of the RD3 C-terminus controls RetGC accumulation in rod outer segments

**Figure 7:**
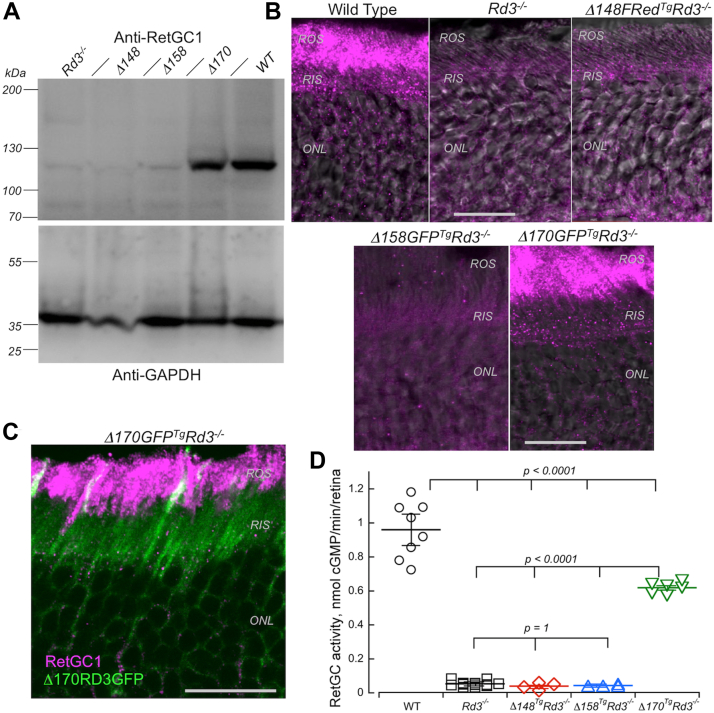
**Restoration of RetGC activity in *Rd3*^*−/−*^ rod outer segments by recombinant RD3 requires its C-terminal region, Arg^158^ – Ser^170^.***A*, extension of the C-terminus of RD3 to Ser^170^ restores RetGC1 accumulation in *Rd3*^*−/−*^; two parts of the same blot were probed with anti-RetGC1 antibody (*top*) or GAPDH (*bottom*). *B*, Anti-RetGC1 immunofluorescence (*magenta* pseudo-color) in wild type, *Rd3*^*−/−*^, *Δ148FRed*^*Tg*^*Rd3*^−/−^, *Δ158GFP*^*Tg*^*Rd3*^−/−^, and *Δ170GFP*^*Tg*^*Rd3*^−/−^; *ROS*, rod outer segments; *RIS*, rod inner segments; superimposed on DIC; bar – 20 μm. *C*, cross-section of *Δ170GFP*^*Tg*^*Rd3*^−/−^ retina with anti-RetGC1 fluorescence (*magenta* pseudo-color) superimposed on Δ170RD3GFP fluorescence (*green*). *D*, RetGC activity measured in the absence of Ca^2+^ at 1 mM free Mg^2+^ in wild type (○), *Rd3*^*−/−*^(□), *Δ148FRed*^*Tg*^*Rd3*^−/−^ (◇, *red diamond*), *Δ158GFP*^*Tg*^*Rd3*^−/−^ (^▵^, *blue triangles*), and *Δ170GFP*^*Tg*^*Rd3*^−/−^ (^▿^, *green triangles*) retinas aged 1 month; the *p* values of the differences are from ANOVA/Tukey HSD test.

The content of main RetGC isozyme, RetGC1, was drastically reduced in *Rd3*^−/−^ retinas (Fig. 7*A*), and it was undetectable in their cross-sections probed by anti-RetGC1 antibody (Fig. 7*B*). Likewise, RetGC1 also remained virtually undetectable in both *Δ148FRed*^*Tg*^*Rd3*^−/−^ and *Δ158GFP*^*Tg*^*Rd3*^−/−^(Fig. 7, *A* and *B*). In contrast, RetGC1 accumulated in *Δ170GFP*^*Tg*^*Rd3*^−/−^ retinas (Fig. 7*A*), where it became strongly detectable in rod outer segments (Fig. 7, *B* and *C*) in a manner similar to wild-type retinas (Fig. 7*B*) or *Rd3*^−/−^ retinas expressing full-length RD3GFP (18).

### RetGC activity in Rd3^−/−^ mice harboring deletion mutants of RD3

*Rd3*^*−/−*^ retinas retain ∼ 70% of rod nuclei at 1 month of age, but the level of endogenous RetGC activity in *Rd3*^*−/−*^ retinal homogenates assayed at low Ca^2+^ concentrations optimal for RetGC activity (32) was almost 20-fold lower (0.05 ± 0.16 nmol cGMP/min/retina) than in wild-type littermates (0.96 ± 0.16 nmol cGMP/min/retina). Although photoreceptor loss at that age was negligible in *Δ148FRed*^*Tg*^*Rd3*^*−/−*^ and *Δ158GFP*^*Tg*^*Rd3*^*−/−*^ mice (Fig. 5), their respective RetGC activities (0.04 ± 0.02 and 0.04 ± 0.004 nmol cGMP/min/retina) remained drastically suppressed in comparison with wild type (ANOVA/Tukey HSD, *p* < 0.0001) and were not significantly different from *Rd3*^*−/−*^ (p ∼ 1). In contrast, RetGC activity in *Δ170GFP*^*Tg*^*Rd3*^*−/−*^ retinas was markedly increased in comparison with *Rd3*^*−/−*^ (*p* < 0.0001) reaching 0.62 ± 0.03 nmol cGMP/min/retina, above half-normal level (Fig. 7*D*).

## Discussion

RD3 in the photoreceptor inner segment (18, 19, 26) plays an essential dual role in photoreceptor physiology and health. A recessive nonsense mutation in the human *RD3* gene causes severe retinal dystrophy and blindness from birth in LCA12 patients (23, 24), similar to the mutation in mouse *Rd3* gene causing blindness in the *rd3* strain (23). The *Rd3*^*−/−*^ genotype in homozygous *rd3* mice results in major deficiencies in their photoreceptors. First, RetGC is not properly delivered to the outer segment (20, 21), which markedly reduces cGMP production in that retinal compartment (22), despite the unaffected availability of GTP as a substrate (33). Consequently, more cGMP-gated CNG channels close in the dark, causing *Rd3*^*−/−*^ photoreceptors to lose their sensitivity to light stimuli. Second, RD3-deficient photoreceptors rapidly degenerate. While the lower-than-normal cGMP production is the reason that the *Rd3*^*−/−*^ photoreceptors become physiologically dysfunctional (Fig. 6), the severity and rapid rate of their degeneration have a different cause. There is likely to be a threshold for lowering cGMP to the levels below which photoreceptors can no longer survive, but the reduced cGMP production in *Rd3*^*−/−*^ photoreceptors cannot explain the fast progression of their death. Even with a complete absence of detectable cGMP synthesis in *RetGC1*,*2*^*−/−*^ retinas (6, 22), their photoreceptors degenerate much more slow than those in *Rd3*^*−/−*^, who still retain a low but detectable level of cGMP synthesis (22). Previous studies indicate that the primary cause of their rapid death is aberrant activation of residual RetGC by GCAPs when the inner segment lacks RD3. Although it is not immediately apparent why photoreceptors do not tolerate this activation, deletion of GCAPs or RetGC1 even further reduces residual RetGC activity in *Rd3*^*−/−*^ photoreceptors but nonetheless rescues the *GCAPs*^*−/−*^*Rd3*^*−/−*^ or *RetGC1*^*−/−*^*Rd3*^*−/−*^ rods from rapid degeneration (18, 19, 29, 33). Evidently, RD3 residing in the inner segment prevents, by uncoupling the GCAP:RetGC complex (Fig. 1 and ref. (18, 22, 26)), the aberrant activation of RetGC and thereby restrains guanylyl cyclase activity until RetGC approaches its proper destination and becomes part of normal phototransduction mechanisms (reviewed in: 1, 12). The results of our study can explain how the structure of RD3 enables its dual function—uncoupling the GCAP:RetGC complex to prevent photoreceptor self-destruction and directing RetGC trafficking from the inner to the outer segment.

Our previous *in vitro* studies identified two clusters of residues that impart to RD3 its high affinity for RetGC, both located in the four-helix bundle core of RD3 (Fig. 1*A*) and not involving the C-terminal half (26, 27). The *Rd3*^*−/−*^ mouse phenotype and LCA12 human blindness result from disruption of RD3 α-helical core (23, 24), which completely disables RD3 binding to RetGC *in vitro* (22). The results of the present study indicate that its α-helical core can by itself suppress fast degeneration of photoreceptors *in vivo*, retaining that function of the normal RD3. RD3 truncated after the α-helical bundle not only retains its ability to bind RetGC1 and uncouple RetGC1:GCAP1 complex *in vitro* (Fig. 1) but also strongly offsets the fast degeneration of *Rd3*^*−/−*^ rods *in vivo*. In both *Δ148FRed*^*Tg*^*Rd3*^*−/−*^ and *Δ158GFP*^*Tg*^*Rd3*^*−/−*^ mice, the majority, over 71%, of the RD3-deficient rods survive even at advanced age (Figs. 4 and 5). However, it should be emphasized that the deficit of RetGC in *Δ148FRed*^*Tg*^*Rd3*^*−/−*^ and *Δ158GFP*^*Tg*^*Rd3*^*−/−*^ outer segment still remains, and their cGMP production is not restored (Fig. 7). Considering the possibility that a severe deficit of cGMP itself is harmful to photoreceptors (26, 34), the complete failure of the two truncated RD3 variants to restore the normal cGMP production may explain why not all *148FRed*^*Tg*^*Rd3*^*−/−*^ and *Δ158GFP*^*Tg*^*Rd3*^*−/−*^ rods survive (Figs. 4 and 5). Restoration of RetGC activity by Δ170GFP RD3 provides the additional ∼20% preservation of rods, almost reaching wild- type.

RD3 truncated at Gly^148^ or Arg^158^ is unable to support RetGC trafficking from the inner to the outer segment (Fig. 7*B*), therefore, rod function cannot be restored in *Δ148FRed*^*Tg*^*Rd3*^*−/−*^ or *Δ158GFP*^*Tg*^ *Rd3*^*−/−*^ (Fig. 6). However, extension of RD3 to Ser^170^ restores rod ERG responses in *Δ170GFP*^*Tg*^*Rd3*^*−/−*^ because Δ170GFP RD3 now dramatically improves RetGC delivery to the *Rd3*^*−/−*^ outer segments, elevating cGMP production to near normal level (Fig. 7*D*).

The difference was particularly striking between Δ170GFP and Δ158GFP RD3 variants. Although the sensitivity of RD3 antibody to the presence of the C-terminal portion of RD3 does not allow for direct quantitative comparison between the two variants, their levels of expression in the retina were comparable (Fig. 3*C*), whereas the ability to restore RetGC was drastically dissimilar (Figs. 4, 6 and 7). Conceivably, the restoration of RetGC levels by Δ170GFP RD3 when it binds to the cyclase could result from better protection of RetGC1 from destruction. However, it is important to emphasize that the entire C-terminal part of RD3 does not participate in binding to RetGC (Fig. 2 and ref. (26, 27)). Both Δ148FRed and Δ158GFP RD3 retain the RetGC binding core and bind RetGC equally or even more efficiently than does Δ170GFP RD3 (Fig. 1*D*). Hence, one should expect that these two variants would protect RetGC at least as efficiently as Δ170GFP RD3, yet they are completely unable to restore the levels of RetGC1 in RD3-deficient rods. A possibility that longer RD3 would better protect RetGC in the complex also seems highly unlikely. Extension of Δ158GFP RD3 by only 12 residues, rather negligible in comparison with the 247 residues of the attached downstream of it ∼28 kDa eGFP, would hardly make such a critical difference in size. Lastly, Δ170GFP, like the other two variants of RD3, retains its cytoplasmic localization and does not accumulate in the nucleus, where it could conceivably affect the RetGC1 gene expression (Figs. 3*F* and 7*C*).

We see as the most reasonable explanation for the results of the present study that Δ170GFP RD3 is recognized by the cellular machinery that delivers membrane proteins and thus allows RetGC to escape into the outer segment where it accumulates. Unlike Δ148FusRed and Δ158GFP, Δ170GFP RD3 evidently regains the ability to properly direct the movement of the RD3:RetGC complex through the cell. Our data strongly indicate that the C-terminal domain of RD3 between Gly^148^ and Ser^170^ is essential for initiating the normal RetGC trafficking and that the R^158^ISPFASDIRTIS^170^ sequence plays a critical role in this process. It would need to be clarified in future experiments if the R^158^ISPFASDIRTIS^170^ presents the entire signal sufficient to direct accumulation of RetGC in the outer segment or requires the whole Gly^148^-Ser^170^ region and/or the RD3 complex with RetGC to enable its function.

**Figure 8:**
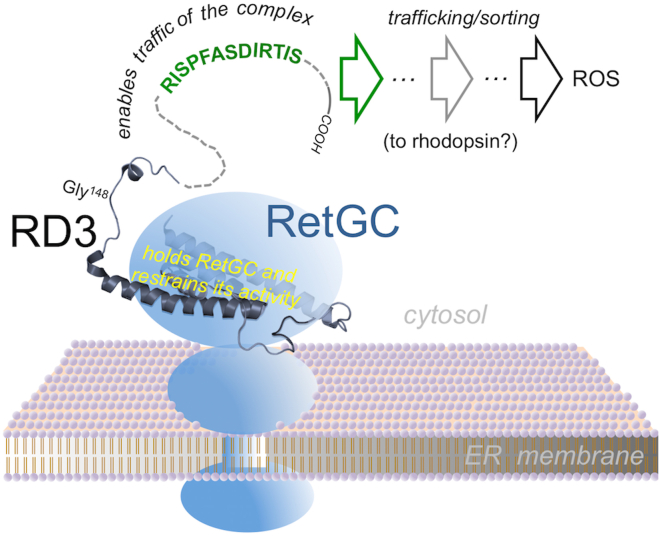
**RD3 structure controls RetGC activity and trafficking: a ‘ski-lift’ hypothesis.** The C-terminal domain of RD3 contains the sequence, R^158^ISPFASDIRTIS^170^, that allows the RetGC:RD3 complex to connect to the path directed toward the outer segment, possibly including as a later step co-transport with rhodopsin-containing membrane vesicles (37). The alpha-helical core of RD3 downstream of Gly^148^ holds RetGC and restrains its activity while in transit through the inner segment, thus preventing aberrant RetGC activation by GCAPs that causes rapid photoreceptor degeneration until the cyclase reaches the outer segment and is released from the complex with RD3 to become regulated by GCAPs (50).

Using a simplified analogy, we propose that RD3 serves as a ‘ski lift’ for RetGC produced in the inner segment (Fig. 8), in which the alpha-helical core of RD3 tightly holds RetGC and restrains its activity in the inner segment, while the C-terminal part of RD3 containing the R^158^ISPFASDIRTIS^170^ signal connects the complex to the trafficking machinery of the cell. Although some of Δ170GFP RD3 can be found in the outer segment, most of it, unlike RetGC, remains in the inner segment, (Figs. 3*B* and 7*C*). To become a functional part of phototransduction, RetGC evidently needs to be released from RD3 upon reaching the outer segment and to form a complex with GCAPs instead. GCAPs compete with RD3 for the cyclase and accumulate in the outer segment with the cyclase (35) restricting RD3 from entering the outer segment (18, 21). GCAPs can also directly displace RD3 from RetGC, although *in vitro* this requires a large excess of GCAPs over RD3 (22, 26). The mutual displacement of RD3 and GCAPs from RetGC may involve complex interactions between them detectable *in vitro* (36), but how these regulatory proteins exchange on their target enzyme *in vivo* remains to be determined.

It is also important to emphasize that RD3 in the model we propose (Fig. 8) only connects the RetGC:RD3 complex to the trafficking machinery. Protein interactions of RD3 that connect the RetGC:RD3 complex to the “cable” of the transporting mechanism and the details of the subsequent trafficking and sorting process would require detailed additional studies. At what point and by what mechanism the membrane compartments containing RetGC and RD3 merge for trafficking and sorting with other membrane proteins remains unclear. There are strong indications that RetGC1 trafficking to the outer segment merges with that of rhodopsin (37). In such a case, the step controlled by RD3 must precede co-trafficking with rhodopsin (Fig. 8), because before *Rd3*^*−/−*^ rods degenerate, they still develop rod outer segments containing rhodopsin, peripherin, CNG1, and PDE6, yet lacking RetGC (20, 21).

## Experimental procedures

### Animals

All experiments involving animals were conducted in accordance with the Public Health Service guidelines and approved by the Drexel University Institutional Animal Care and Use Committee. The wild type C57BL/6J and *rd3* mouse strain originated from JAX Research/Jackson’s Laboratory. The *rd3* mice were made congenic with the C57BL/6J genetic background by breeding for over 15 generations prior to conducting the experiments. Mice were fed the same diet and were housed in the same temperature- and humidity-controlled environment using 12 h/12h light/dark cycle. Transgenic mice harboring RD3 constructs under 4.2 kb rod opsin promoter were produced using random insertion of the constructs injected in male pronuclei of fertilized C57B6J mouse eggs (service provided by Cyagen) as previously described for RD3GFP (18). The founders were selected by testing fluorescence of the tagged proteins in mouse retinas. The expressing lines developed in C57B6/J background were outcrossed to rd3/rd3 mice to produce *Δ148FRed*^*Tg*^*Rd3*^*−/−*^ and *Δ170GFP*^*Tg*^ *Rd3*^*−/−*^. The presence of the integrated DNA constructs was detected using PCR of mouse tail DNA samples utilizing specific for the constructs oligonucleotide primers, and the expression of the proteins by the transgenes in the established lines was verified by fluorescence microscopy. The *Rd3*^*−/−*^ genotype was verified by PCR as previously described (18, 26).

### RD3 constructs

DNA fragments coding for the fluorescently tagged deletion mutants of human RD3 were amplified from the previously characterized RD3GFP construct (18) using high-fidelity PhusionFlash DNA polymerase (ThermoFisher) or chemically synthesized (Integrated DNA Technologies). To produce GFP-tagged deletion mutants, the RD3 cDNAs were inserted into pET11d (Novagene/Calbiochem) vector for bacterial expression, modified pRCCMV vector (Invitrogen) for expression in HEK293 cells, or pBluescript vector harboring rod opsin promoter and polyadenylation signal for transgenic expression in mouse rods, all containing previously described RD3GFP cDNA (18), to replace the full-length RD3 sequence in the fusion protein with those of the deletion mutants. The FusionRed (28) cDNA for Δ148FRed RD3 was chemically synthesized (Integrated DNA Technologies) and used to replace the eGFP cDNA in the Δ148GFP fusion construct.

### Expression and purification of GCAP and RD3

Bovine GCAP1 was expressed from pET11d vector in a BLR(DE3) *E*. *coli* strain (both originated from Novagen/Calbiochem) harboring pBB131 plasmid coding for a yeast N-myristoyl transferase, and the expressed myristoylated GCAP1 was extracted by urea from inclusion bodies, refolded and purified using hydrophobic and size-exclusion chromatography as previously described (38, 39) to the purity of recombinant estimated by SDS gel electrophoresis ≥ 90%. Human RD3GFP and its tagged deletion mutant variants for *in vitro* assays were expressed from pET11d vector in a BLR(DE3) *E*. *coli* strain and purified by urea extraction/precipitation and centrifugation as previously described (22, 26). Protein concentrations were determined as described (26) by 280 nm absorbance in 50 mM Tris-HCl (pH 7.5) containing 7 M guanidine chloride, assuming 0.1 g/L absorbance calculated using a ProtParam software (ExPASy server).

### RetGC activity assays

Recombinant RetGC activity in HEK293 cells transfected with a human RetGC1 cDNA was measured under ambient illumination using a crude membrane fraction isolated from the transfected cells as previously described in detail (39, 40). Endogenous RetGC activity in mouse retinas was assayed as previously described in detail (18, 41, 42). The retinas for the assay were excised using a microscope fitted with infrared goggles from mice dark-adapted overnight and euthanized under infrared illumination, frozen in liquid N_2_ and stored at −70 °C. The assays were conducted under infrared illumination. RetGC assay mixtures (25 μL) containing HEK293 membranes or retinal homogenates in 30 mM MOPS–KOH (pH 7.2), 60 mM KCl, 4 mM NaCl, 1 mM DTT, 2 mM EGTA, 1 mM free Mg^2+^, 0.3 mM ATP, 4 mM cGMP, 1 mM GTP, and ∼1 μCi of [α–^32^P]GTP, 100 μM zaprinast and dipyridamole, and 10 mM creatine phosphate/0.5 unit of creatine phosphokinase (Sigma-Aldrich/Millipore) were incubated at 30 °C for 40 min in case of HEK293 cell membranes or 12 min in case of retinal homogenates, and the reaction was stopped by heat-inactivation at 95° for 2 min. The [^32^P]cGMP product separated by TLC on fluorescently-backed polyethyleneimine cellulose plates (Merck) in 0.2 M LiCl was eluted and counted using liquid scintillation. The retinal assay contained ∼0.5 μCi [^3^H]cGMP as the internal standard to ensure the lack of the cGMP product hydrolysis by phosphodiesterase 6. Data fitting and analysis was performed using a Synergy Kaleidagraph 4 software.

### Cell-based RD3/RetGC1 co-localization assay

The assay was conducted as originally described in detail in (43) with modifications subsequently described in (44, 45). In brief, HEK293 cells were transfected in LabTeck 4-well cover glass chamber with 1 μg of mOrangeRetGC1 DNA per well using 3 μL/μg DNA of the Promega FuGENE reagent following the protocol recommended by the manufacturer at ∼1:100 molar ratio of RD3GFP coding plasmids *versus* mOrangeRetGC1 coding plasmid or a control plasmid not containing RetGC1 cDNA, ∼1 μg total DNA/well. Confocal images were typically taken after 24 to 32 h of incubation in 5% CO_2_, 37 °C, utilizing an Olympus FV1000 Spectral instrument with the respective 543 nm and 488 nm excitation wavelengths for the red and the green fluorochromes. Images collected in a sequential mode were superimposed on differential interference contrast (DIC) and processed using Olympus FluoView FV10-ASW software. No changes to the original images were made except for minor gamma correction applied to the whole image for more clear presentation in print. When pertinent, the fluorescence was assigned color-blind-friendly pseudo colors.

### Optical coherence tomography (OCT)

Mice were anesthetized using intraperitoneal injection of 20 mg/kg Ketamine and 8 mg/kg Xylazine (Dechra). The pupils were dilated by applying 1% Tropicamide and 2.5% Phenylephrine ophthalmic eye drops (Bausch and Lomb, Inc.) 5 to 10 min before the scan. The A- and then B-scans of the retinas were acquired using an iiScience spectral-domain OCT camera calibrated by the manufacturer at 2.47 μm/pixel axial scale and 3.5 μm/pixel lateral scale resolution and typically averaged from ∼200 frames using National Institutes of Health ImageJ/Fiji software as described in (43). The thickness of the ONL layer was measured between the outer plexiform and the external limiting membrane reflective layers (46, 47) in the B-scans across the retina ∼ 400 to 700 μm below the optic located by the A-scan.

### Retinal histology

Mice anesthetized by intraperitoneal injection of Ketamine/Xylazine (180/48 mg/kg) were perfused through the heart with phosphate-buffered saline (PBS), followed by 3.5% paraformaldehyde and 2.5% glutaraldehyde in PBS. The eyes were processed for paraffin embedding, sectioned, and stained with hematoxylin/eosin (service by AML Laboratories, Saint Augustine, FL) as previously described (43). The stained sections were photographed between the optic nerve and the periphery of the retina using an Olympus Magnafire camera mounted on an Olympus BX21 microscope.

### Electroretinography (ERG)

Mice aged 1 to 1.5 months were dark adapted overnight, then 1% Tropicamide and 2.5% Phenylephrine ophthalmic eye drops were applied to dilate the pupil under dim red safelight illumination, and the mice were dark-adapted for another 10 min. Full-field ERG in mice anesthetized by inhalation of 1.7 to 1.9% Isoflurane (VEDCO)/air mix delivered at the rate 50 ml/min by a Kent Scientific SomnoSuite setup was performed in the dark using a Phoenix Research Laboratories Ganzfeld ERG2 instrument. Light pulses (505 nm, 1 ms) producing ∼0.5 × 10^6^ photoisomerized rhodopsin (R^∗^) molecules per rod were injected through the infrared camera-guided corneal electrode combined with the LED light source of the instrument calibrated by the manufacturer.

### *Antibodie*s

Anti-RetGC1 (RRID:AB_2877058) rabbit polyclonal antibody (48) (used at dilution 1:10000) and anti-human RD3 rabbit polyclonal antibody 497 (22) (used at dilution 1:4000) were characterized previously and validated using transgenic mouse models (6, 18, 35). Rabbit polyclonal GAPDH antibody (used at dilution 1:4000) was purchased from OriGene. A Pierce goat anti-rabbit peroxidase-conjugated antibody for immunoblotting (used at dilution 1:20000) and Molecular Probes AlexaFluor 543 goat anti-rabbit antibody for immunofluorescence microscopy (used at dilution 1:350) were purchased from Fisher Scientific.

### Immunofluorescence

Mice were euthanized by lethal injection of Ketamine/Xylazine and then perfused through the heart, first with PBS and then with 5% formaldehyde in PBS. Enucleated eyes were processed and embedded for cryosectioning as previously described (18, 43). The cryosections were probed with the rabbit anti-RetGC1 antibody (diluted 1:10000 in PBS containing 5% bovine serum albumin (Millipore/Sigma), goat serum and 0.1% Triton X-100. The signal was developed using AlexaFluor 543 antibody diluted 1:350 in the same solution. The confocal imaging of fluorescence superimposed on DIC was performed using an Olympus FV1000 Spectral instrument.

### Immunoblotting

The retina homogenates were subjected to extraction in AbCam Radio Immuno Protein Assay mixture of detergents supplemented with protease inhibitors cocktail (Millipore/Sigma). The samples mixed with 1:1 volume of 2×Laemmli SDS sample buffer (Millipore/Sigma) were subjected to electrophoresis in 4 to 12% (for RetGC1) or 4 to 20% (for RD3 constructs) polyacrylamide gel (PAAG). The proteins were transferred overnight at 40V constant voltage to Immobilon P membrane (Millipore) at 18 °C using Tris-glycine transfer buffer (Fisher Scientific). SuperBlock solution in TTBS buffer (ThermoScientific) containing 5% goat serum (Millipore/Sigma) was used for blocking the membrane and for antibody dilution. The luminescence signal was developed using a Pierce peroxidase-conjugated goat anti-rabbit antibody and SuperSignal Femto substrate reagent (ThermoScientific). The images were acquired and processed using a Fotodyne Luminous FX imager and ImageJ/Fiji software (National Institutes of Health). Where indicated, the same Immobillon P membrane was then washed three times for 5 min in H_2_O, stripped from the RD3 detection system by boiling for 5 min in 10 mM TrisHCl (pH 7.4) containing 5% mercaptoethanol and 2% SDS, washed in H_2_O 10 times × 5 min, and re-probed for GAPDH.

### Statistics

Where applicable, Kolmogorov-Smirnov or Shapiro-Wilk tests were applied to the data and statistical significance of the differences was evaluated by one-way ANOVA with Tukey HSD all-pairs comparison *post hoc* test (alpha = 0.01) using a Synergy Kaleidagraph software. The pertinent statistical differences are presented in Results.

*RD3 structural model* (6drf.pdb) (25) was presented using the Schrödinger PyMol software.

### Data availability

The data referred to in this manuscript are contained within the manuscript. Unprocessed data can be available upon reasonable request from the corresponding author (alexander.dizhoor@drexel.edu).

## Conflict of interest

The authors declare that they have no conflicts of interest with the contents of this article.
